# Validation of the London Measure of Unplanned Pregnancy among pregnant Australian women

**DOI:** 10.1371/journal.pone.0220774

**Published:** 2019-08-08

**Authors:** Adina Y. Lang, Jennifer A. Hall, Jacqueline A. Boyle, Cheryce L. Harrison, Helena Teede, Lisa J. Moran, Geraldine Barrett

**Affiliations:** 1 Monash Centre for Health Research and Implementation, School of Public Health and Preventive Medicine, Monash University, Melbourne, Victoria, Australia; 2 Research Department of Reproductive Health, UCL EGA Institute for Women’s Health, University College London, London, United Kingdom; Liverpool School of Tropical Medicine, UNITED KINGDOM

## Abstract

**Introduction:**

Globally, over half of pregnancies in developed countries are unplanned. Identifying and understanding the prevalence and complexity surrounding pregnancy preparation among Australian women is vital to enable sensitive, responsive approaches to addressing preconception and long-term health improvements for these women with varying motivation levels.

**Aim:**

This study evaluated the reliability and validity of a comprehensive pregnancy planning/intention measure (London Measure of Unplanned Pregnancy) in a population of pregnant women (over 18 years of age) in Australia.

**Methods:**

A psychometric evaluation, within a cross-sectional study comprising cognitive interviews (to assess comprehension and acceptability) and a field test. Pregnant women aged over 18 years were recruited in early pregnancy (approximately 12 weeks’ gestation). Reliability (internal consistency) was assessed using Cronbach’s alpha, corrected item-total correlations and inter-item correlations, and stability via a test-retest. Construct validity was assessed using principal components analysis and hypothesis testing.

**Results:**

Six women participated in cognitive interviews and 317 in the field test. The London Measure of Unplanned Pregnancy was acceptable and well comprehended. Reliability testing demonstrated good internal consistency (alpha = 0.81, all corrected item-total correlations >0.20, all inter-item correlations positive) and excellent stability (weighted kappa = 0.92). Validity testing confirmed the unidimensional structure of the measure and all hypotheses were confirmed.

**Conclusions:**

The London Measure of Unplanned Pregnancy is a valid and reliable measure of pregnancy planning/intention for the Australian population. Implementation of this measure into all maternity healthcare, research and policy settings will provide accurate population-level pregnancy planning estimates to inform, monitor and evaluate interventions to improve preconception health in Australia.

## Introduction

Global estimates report that over half of all pregnancies in high income countries are unplanned [1]. Unplanned pregnancies are associated with suboptimal reproductive healthcare engagement and preconception health behaviors, as well as a range of adverse maternal and neonatal outcomes including pregnancy loss, low birth weight and neonatal mortality [2–5]. Conversely, planning for pregnancy is predictive of positive behavior change such as preconception smoking cessation and commencement of folic-acid and multivitamin supplementation [6].

The preconception period, 2–3 months prior to pregnancy [5], represents a critical time in which the health behaviors of reproductive-aged women and men can influence their general wellbeing, reproductive health, pregnancy outcomes and the health of their family and future generations [7]. These behaviors include: changing eating practices and micronutrient supplementation aligned with national guidelines; undertaking regular physical activity; achieving a healthy weight; smoking, recreational drug and alcohol cessation; chronic disease management; health screening; and updating immunizations [8]. Yet limited awareness of preconception health remains a key barrier to engagement in these healthy behaviors in both women and men [5]. This contributes to a missed opportunity to optimize health in preparation for pregnancy that benefits women’s own wellbeing and that of their child [8]. In addition, prior studies have shown that women experiencing socio-economic disadvantage, low educational attainment, unemployment, who are <25 years of age, single or overseas-born are more likely to experience unplanned pregnancies [9–11]. Identifying and more deeply understanding the detail surrounding pregnancy planning and preparation will enable a more targeted and tailored approach to addressing preconception health improvements for women with varying levels of planning, health risks and motivations.

In Australia, 310,247 women gave birth in 2016 [12]. The average age of all these women was 30.5 years, 29.0 years for first-time mothers and most mothers were in a marital relationship (66%). Nearly all births (97%) occurred in hospitals, most commonly in public hospital settings (74%). Whilst abortion is legal (or decriminalized) throughout Australia, abortion laws vary between different states [13]. Information available on abortion rates is limited, however, recent data collected in the state of South Australia estimated induced abortion rates of 13.5 per 1,000 women aged 15–44 years in 2015, with most of these terminations performed in metropolitan public hospitals [14]. Research has described that approximately half of women reporting unplanned pregnancies choose to parent, approximately one-third choose abortion, a few (2%) opt for adoption and 13% of women experience a miscarriage [15].

Pregnancy planning and intention is a complex multifaceted concept and an unplanned pregnancy may not mean an unwanted pregnancy [16]. To date, Australian studies have used a variety of measures to assess pregnancy planning [9, 11] including the London Measure of Unplanned Pregnancy (LMUP) [17, 18]. The LMUP is a comprehensive measure of pregnancy intention which aims to accurately capture the nuances in behaviors and feelings surrounding conception [19]. However, it is yet to be validated in the Australian context. To address this gap, the current study adapts the LMUP and evaluates its suitability, reliability and validity to assess pregnancy planning in a population of pregnant women living in Australian.

## Methods

To evaluate the LMUP’s psychometric properties in Australia, Classical Test Theory item analysis, consistent with prior LMUP evaluation methodology [19], was employed. The LMUP evaluation was embedded within a cross-sectional study questionnaire examining pregnancy intention and preparation behaviors.

### The instrument—LMUP

The LMUP is a self-administered retrospective measure including six items (questions) assessing the extent of pregnancy planning/intention of current or recent pregnancies [19]. Originally developed in the United Kingdom (UK), the tool has been validated across different countries and in several language versions [20–27]. The items relate to contraceptive use, pregnancy timing, intentions, desire, partner influence and preparation. A score of zero, one or two can be achieved for each item (Table 1 provides a summary of LMUP items and response options). Scores are combined to calculate an overall pregnancy intention score ranging from zero to 12, with increasing scores representing higher degrees of planning/intention. Whilst the scale is best used in a continuous form, for population estimates the scores can be divided into three classifying groups: 10–12 (planned), 4–9 (ambivalent); and 0–3 (unplanned) [19]. If a binary score for the LMUP is used, scores should be classified as ≥10 being planned and 0–9, unplanned [28].

**Table 1:** Frequencies of LMUP items and response options (n = 317).

Item	Response options	Scores	
		n	%
1. Contraception	0, always used contraception	4	1.3
	1, used sometimes or failed at least once	27	8.5
	2, did not use contraception	286	90.2
2. Timing	0, wrong time	3	0.9
	1, ok, but not quite right time	72	22.7
	2, right time	242	76.3
3. Intention	0, did not intend pregnancy	37	11.7
	1, intentions kept changing	40	12.6
	2, intended pregnancy	240	75.7
4. Desire	0, did not want baby	8	2.5
	1, mixed feelings about having baby	53	16.7
	2, wanted a baby	256	80.8
5. Partner	0, never discussed getting pregnant	5	1.6
	1, discussed but did not agree to get pregnant	40	12.6
	2, agreed to get pregnant	272	85.8
6. Preparation	0, no health actions	90	28.4
	1, one health action	59	18.6
	2, 2 or more health actions	168	53.0

#### Adaptation of the LMUP for the Australian context

Prior to cognitive interviews (described below) two response options were added to item six relating to pregnancy preparation including whether women 'took iodine' or 'took vitamin D' (see Australian LMUP, http://measure.ascody.co.uk/lmupaustralia.htm). Iodine was added in accordance with recommendations by the Australian National Health and Medical Research Council [29] and the Australian general practice preconception care guidelines [30]. Consideration of vitamin D deficiency was also clinically relevant at the time as women’s levels were tested as part of routine antenatal care in the state of Victoria. The addition of these options did not affect the overall scoring.

### Settings, recruitment and participants

Women were recruited in early pregnancy through Monash Health maternity services, a large public maternity service in Victoria, Australia and through the member database of one of the largest Australian national private health insurers, Medibank Private Limited (Medibank).

Monash Health is one of the largest Australian healthcare services, with approximately 9,000 births in 2017–18 across the catchment, catering to a diverse population including 62% of women born overseas. In Australia, women have the option to pay for private healthcare in pregnancy; and while 74% of hospital births in Australia occur in public hospitals, many women (26%) elect to give birth in private hospitals [12]. To enable the inclusion of a broad national cross-section of reproductive age women, Medibank, one of the principal private healthcare providers in Australia providing obstetrics cover for approximately 20,000 Australian births annually, was also engaged in the study.

Prior to the study’s commencement, A.Y.L, C.L.H and L.J.M met with and presented the study to the clinic midwives and staff to establish an appropriate recruitment process at the public maternity service. A collaboration was established with the private health insurer and the recruitment process was co-developed with members of the research team (C.L.H, J.A.B, and H.T).

#### Ethics

Ethics approval for this study was granted by the Monash Health (RES-17-0000-087A) and Monash University (Project no. 10370) Human Research Ethics Committees. All women provided informed consent.

#### Monash health—Public maternity service

A study invitation flyer was mailed to all women attending the public maternity service clinic over the study period (August 2017 to March 2018) before their first antenatal appointment. Women were eligible to take part if they were pregnant, aged over 18 years, attending the public maternity services for their first antenatal visit or who received the invitation flyer and expressed interest in participating prior to their first maternity appointment. Women were excluded if they were unable to communicate or comprehend sufficiently in English.

A secure link to complete the questionnaire online or a hard copy was provided. As gratitude, in recognition of participation, all women at the public maternity service were placed in the draw to receive a $100AUD gift card.

#### Medibank—Private health system

Eligible women were private health insurance members, aged 18–40 years who had taken out, or upgraded their private health cover to include obstetrics within the 12 months prior to recruitment commencement (January 2018), and who self-identified as being pregnant. The eligible age range was defined by the health insurer.

A database of eligible members was provided by the health insurer. A co-developed invitation for participation was emailed to all eligible women explaining the voluntary nature of participation. Women opted-in to the study providing implied consent by following the link and completing the online questionnaire.

### Phase 1: Pre-testing—Cognitive interviews

Cognitive interviews [31] were conducted through the public maternity service to pre-test the LMUP. Women completed the questionnaire, followed by the interview where the research used probing techniques to determine appropriateness and comprehension of the questions by women. There is no specified minimum number of interviews required for testing a questionnaire, however, small numbers have been found to be sufficient, with a maximum of 12–15 interviews recommended [32]. These interviews were conducted by the research lead (A.Y.L) as individual face-to-face or telephone sessions which ran for approximately 30 minutes. Detailed notes were taken and any changes were discussed with members of the research team. Initial testing and re-testing was conducted. No concerns were raised by women regarding the comprehension of the LMUP questions during interview and no changes were made.

### Phase 2: LMUP–Validity and reliability testing

Following recruitment for cognitive interviews, women recruited into the main study consented to complete the initial LMUP (embedded within the broader cross-sectional study questionnaire) and a repeat of the LMUP only (online or hard copy) was sent two weeks later to assess test-retest reliability. Recruitment for the test-retest cohort ceased once sufficient numbers (~100) had been reached [33, 34]. The overall sample size exceeded minimum recommendations for validation studies [35]. The remaining women completed the LMUP embedded within the questionnaire at one time point only.

#### Demographic information

Self-reported demographics were collected. Age was calculated from women’s date of birth. Women were asked if they had any children and if so, how many. Relationship status at 3 months before pregnancy was asked and included the following response options: married, defacto, never married, widowed, divorced, separated. Place of birth was asked with response options including: Australia, United Kingdom, India, Sri Lanka, New Zealand, Afghanistan, Philippines, Mauritius, China, South Africa or other. Women were asked to indicate their main employment status in the year before pregnancy as: full time paid work, part time or casual paid work, work without pay [e.g. in a family business], home duties only–no paid work, studying, unemployed–looking for work, unpaid voluntary work, unable to work due to sickness or injury or other. Women provided their postal code and were asked to indicate their highest level of education (response options included: no post-school qualification, Certificate III/IV, Advanced Diploma and Diploma, Bachelor Degree, Graduate Diploma and Graduate Certificate or Postgraduate Degree [e.g. Masters, PhD]). Response categories were combined for analysis (see Table 2). Additional information was obtained from the public hospital medical records including: women’s gestation at first visit, gravida, parity, previous birth outcomes and postal code. A woman’s socio-economic status (SES) was estimated by aligning her postal code with a corresponding score (decile) defined by the Australian Socio-Economic Indexes for Areas (SEIFA), Index of Relative Socio-Economic Disadvantage [36]. SEIFA deciles were estimated based on a combination of variables from the National Census e.g. income, education and unemployment. An area (determined by postal code) receives a score (deciles) ranked on a scale of disadvantage relative to other areas. Deciles 1–3 were classified as low SES, 4–7 middle SES and 8–10 high SES. SEIFA is used as standard indicator of SES in Australia making it comparable with other studies [17].

**Table 2:** Characteristics of women completing the LMUP compared to the national antenatal populations.

	Total n = 317	LMUP test-retest (twice) n = 131	LMUP non-retest (once only) n = 186	Comparison of LMUP test-retest and non-retest groups	National pregnant population^
**Age (years)**	n = 284	n = 131	n = 153	P = 0.95	
Mean (SD)*	30.1 (4.7)	30.1 (4.6)	30.1 (4.8)		30.5
	***n (%)***	***n (%)***	***n (%)***		***n (%)***
**Age group**	n = 284	n = 131	n = 153	P = 0.16	
*<25*	36 (12.7)	17 (13.0)	19 (12.4)		44,816 (14.5)
*25–29*	91 (32.0)	36 (27.5)	55 (35.9)		83,667 (27.0)
*30–34*	107 (37.7)	58 (44.3)	49 (32.0)		110,946 (35.8)
*>35*	50 (17.6)	20 (15.3)	30 (19.6)		70,776 (22.8)
***Children***	n = 287	n = 131	n = 156	P = 0.70	
*0–2*	270 (94.1)	124 (94.7)	146 (93.6)		n/a
*≥3*	17 (5.9)	7 (5.3)	10 (6.4)		n/a
**Relationship status**	n = 273	n = 131	n = 142	P = 0.51	
*Married/ De facto*	255 (93.4)	121 (92.4)	134 (94.4)		N/A (66 married) [40]
Unmarried**	18 (6.6)	10 (7.6)	8 (5.6)		n/a
**Place of birth**	n = 273	n = 131	n = 273	P = 0.74	
*Australia*	157 (57.5)	74 (56.5)	83 (58.5)		201,984 (65.1)
*Outside Australia*	116 (42.5)	57 (43.5)	59 (41.5)		106,572 (34.4)
**Previous live birth**	n = 287	n = 131	n = 156	P = 0.01	
*Yes*	148 (51.6)	78 (59.5)	70 (44.9)		176,671 (56.8)
*No*	139 (48.4)	53 (40.5)	86 (55.1)		132,842 (42.8)
**Education**	n = 273	n = 131	n = 142	P = 0.43	
*Post-secondary school*	228 (83.5)	107 (81.7)	121 (85.2)		n/a
*School only*	45 (16.5)	24 (18.3)	21 (14.8)		n/a
**Employment**	n = 273	n = 131	n = 142	P = 0.83	
*Paid employment*	211 (77.3)	102 (77.9)	109 (76.8)		n/a
*Unpaid employment/ unemployed*	62 (22.7)	29 (22.1)	33(23.2)		n/a
**Public/ private healthcare**	n = 317	n = 131	n = 186	n/a	
*Private*	92 (29.0)	n/a	92 (49.5)		26%
*Public*	225 (71.0)	131 (100)	94 (50.5)		74%
**SIEFA**	n = 283	n = 131	n = 152	P = 0.74	
*Higher level disadvantage (Decile 1–5)*	87 (30.7)	39 (29.8)	48 (31.6)		n/a
*Lower level disadvantage (Decile 6–10)*	196 (69.3)	92 (70.2)	104 (68.4)		n/a

Note: Total *n* may vary due to missing data.

^Supplementary tables for Australia's mothers and babies 2016—in brief [12].

*Data for age was reported as mean (standard deviation), all other data were presented as frequencies (percentages) and analyzed using the student’s t-test or chi-square.

**Never married/ widow/ divorced /separated.

Abbreviations: LMUP = London Measure of Unplanned Pregnancy, SIEFA = Socio-Economic Indexes for Areas, SD = Standard deviation.

### Statistical analysis

Data analysis was performed following the Classical Test Theory-based approach using SPSS version 25 (Armonk, New York) and STATA version 14 (College Station, Texas).

#### Acceptability and targeting

Acceptability of measure was initially examined through cognitive interviews and further determined by assessing missing data rates, with lower rates (<5%) indicating greater acceptability [37]. Distribution of total LMUP scores were checked to examine whether the full range of scores had been captured as an indication of targeting of the measure. The percentage of women that selected each response option for an item (item-endorsement) was examined to provide information about item discrimination [38].

#### Reliability

Reliability (internal consistency) was examined using the Cronbach’s alpha (α) statistic (where a score >0.7 was used to indicate acceptable reliability) and corrected item-total correlations (where <0.2 indicates items contribute little to the homogeneity of the scale) [38]. Inter-item correlations were also examined to check that they were positively correlated. Test-retest reliability was measured with a sub-sample of the population using the weighted kappa (κ) to reflect the stability of women’s scores. Agreement scores were classified as moderate (0.41–0.60) or substantial (0.61–0.80), to near perfect (0.81–1.00) [39].

#### Validity

Construct validity was evaluated by principal component analysis (PCA) and hypothesis testing. PCA assessed the internal structure of the LMUP by testing whether all items loaded onto one component (unrotated, with an Eigenvalue >1), demonstrating that the LMUP is measuring a single underlying construct. To test whether the LMUP behaved as it should, hypotheses supported by prior pregnancy intention literature and LMUP validation studies were tested [9, 17, 19, 20]. Based on prior literature, it was hypothesized that: LMUP scores will be lower (more unplanned) in women with more live children (≥3) and women who were not in a relationship (married or de facto) [9, 17, 19, 20]. Continuous variables were presented as mean± standard deviation (SD) or median and inter-quartile range (IQR) as appropriate. Categorical variables were presented as frequencies (percentages). The student’s t-test or chi-square was used as appropriate to examine any differences between demographics of women who completed the retest and those that did not and those in the public and private groups. As LMUP scores followed a non-parametric distribution, the Mann Whitney U test was used to assess hypotheses. P values <0.05 were considered statistically significant. Criterion validity, comparing newly developed psychometric tools with established ‘gold standard’ measures of the same construct, was not possible due to the absence of prior tools [19].

## Results

### Samples and characteristics of participating women

Cognitive interviews were conducted with six pregnant women recruited through the public maternity service. The mean±SD gestation of these women at recruitment (first antenatal visit) was 12.5±1.1 weeks, with a mean age of 33.5±1.4 years. All six women were in a relationship (married n = 5, de facto n = 1), all had children and a post-secondary school education and three were Australian born. In the year before pregnancy, four of these women were unemployed.

**Fig 1:**
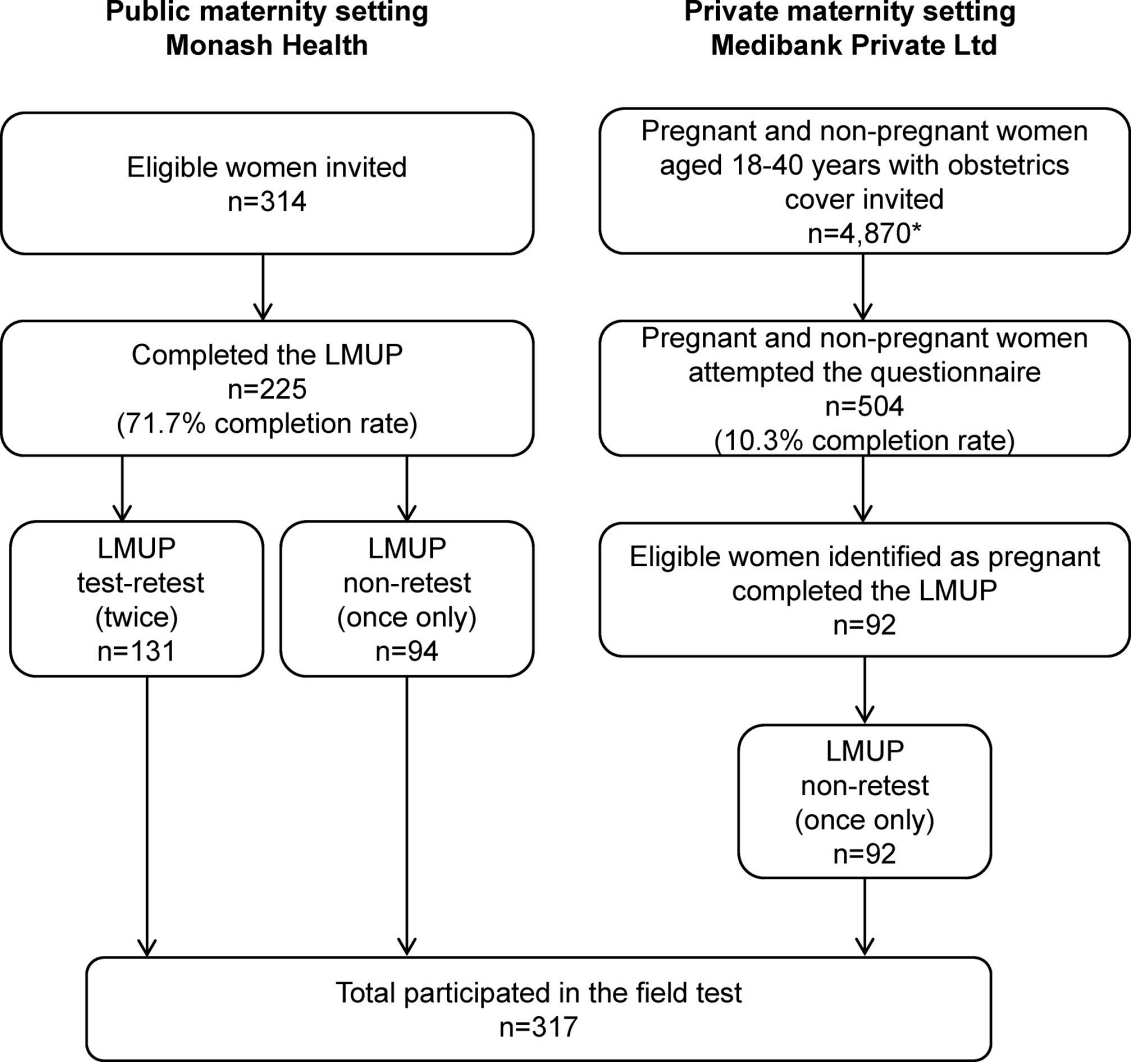
Recruitment and participation flow diagram. *56.8% of all invited women (n = 4,870) opened the original email invitation, 13.5% opened the questionnaire.

The LMUP field test was completed by 317 pregnant women in the public (n = 225) and private (n = 92) maternity care systems. Of the women from the public hospital cohort only, 314 women were eligible and initially recruited to participate in the study (225 of these women in the public hospital who agreed to participate completed the questionnaire, a 71.7% completion rate), 131 of which also completed the test-retest component. Women recruited through the private health insurer completed the embedded LMUP once only. An initial email was sent to eligible women in the Medibank database (n = 4,870 including both pregnant and non-pregnant women), 56.8% of these women opened the original email invitation, 13.5% opened the questionnaire and 10.3% (n = 504) attempted the overall questionnaire. For the purpose of this study only women who identified as being pregnant and who completed the LMUP questions were included, of which there were 92. See participation flow diagram, Fig 1.

The mean±SD gestation of women from the public maternity service at the time of recruitment (first antenatal visit) was 8.12±3.7 weeks. Data were not available for women recruited through the private health insurer. Overall, the mean±SD age of women was 30.1±4.7 years. The majority (93.4%) of women were married (70.3%, n = 192) or de facto (23.1%, n = 63), had two children or fewer (94.1%, n = 270) and two-fifths were born overseas (42.5%, n = 116). The sample had high levels of post-secondary school education (83.5%, n = 228) and paid employment in the year before pregnancy (77.3%, n = 211) (Table 2). Overall, data were consistent with national averages (Table 2).

Demographic differences between the public and private cohorts included age (public 29.8±4.9 years vs private 31.2±4.0 years, p = 0.04), education (post-school qualification; public 79.2% vs private 98.4%, p<0.001) and employment in the year before pregnancy (employed; public 73.6% vs private 90.2%, p = 0.01). There were no differences in relationship status, number of children, place of birth and socio-economic status.

The LMUP retest was completed by a subset of these women recruited through the public maternity service (n = 131). The only demographic difference among the retest and non-retest groups was whether they had a previous live birth (previous live birth; 59.5% retest vs 44.9% non-retest, p = 0.01), there were no other significant demographic differences between these groups (Table 2).

### Acceptability and targeting

Cognitive testing of the LMUP did not reveal any concerns regarding the comprehension of the original LMUP items and no further changes were made. The lack of missing data in women’s responses in the field test, triangulated with the findings from cognitive interviews, suggest that the items were acceptable to women.

**Fig 2:**
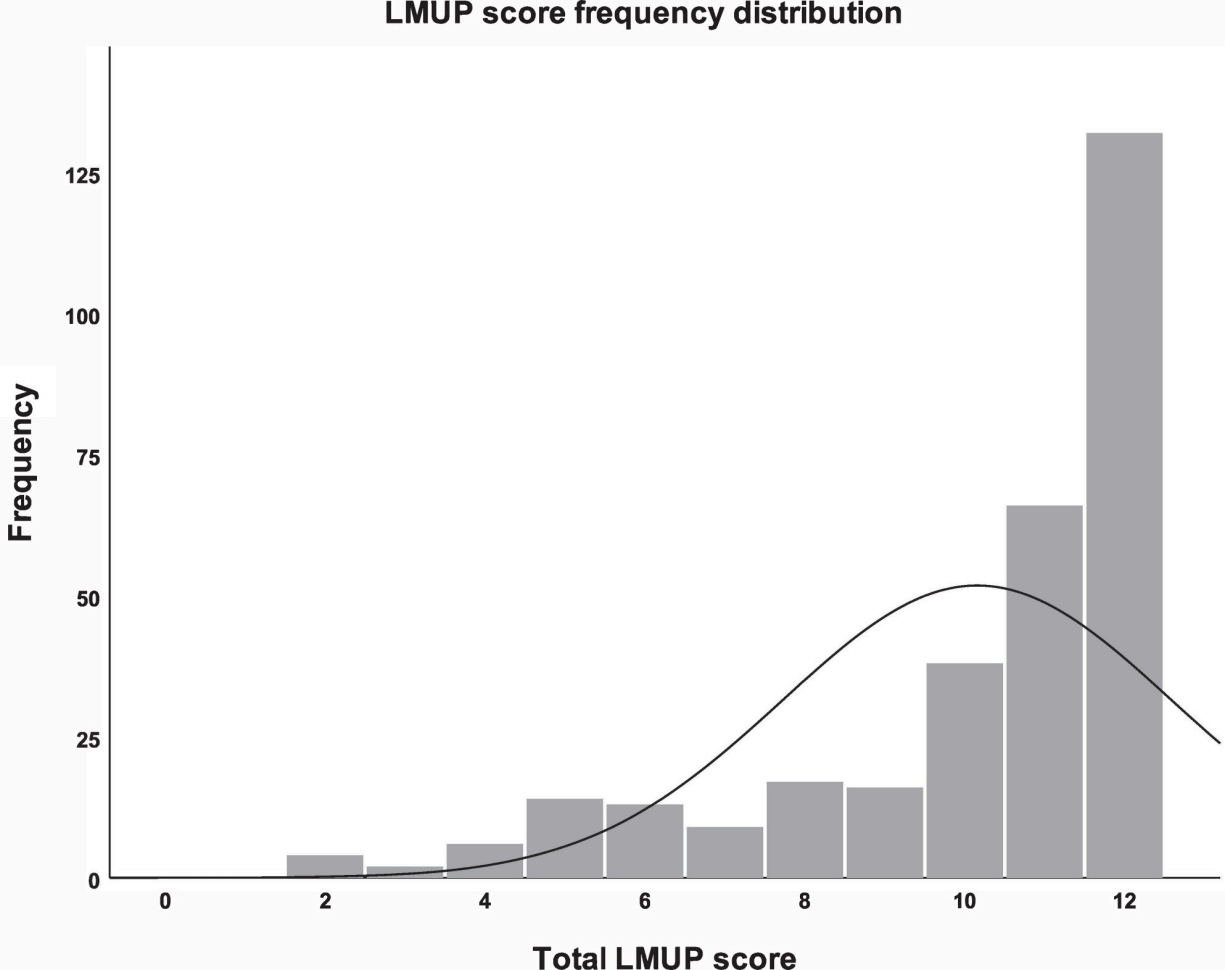
Distribution of LMUP scores.

Total LMUP scores ranged from 2 to 12. No woman received a score of 0 or 1 and therefore the score distribution was left-skewed (Fig 2). The median (IQR) score for all women was 11 (9, 12). Overall 74.4% of all women had a score of 10–12 (planned), 23.7% scored 4–9 (ambivalent) and 1.9% scored 0–3 (unplanned). In terms of item response options, the highest endorsements were “I/we were not using contraception” in item 1, “My partner and I agreed that we would like me to be pregnant” in item 5, and “I wanted to have a baby” in item 4 (Table 1).

### Reliability

The Cronbach’s α was 0.81, the corrected item-total correlations were all >0.2 (item 1 0.42, item 2 0.66, item 3 0.78, item 4 0.64, item 5 0.65, item 6 0.53) and inter-item correlations were all positive.

The average number of interval days between test-retest was 17±5.7 days. The Cohen’s Kappa was 0.92 and Percent Agreement was 0.99. The median (IQR) LMUP scores remained the same at test and retest, 11 (9, 12).

### Validity

Principal component analysis confirmed the unidimensional structure of the scale, with one component (Eigenvalue, 3.4) and all six items loading onto that component (item 1 0.55 item 2 0.80 item 3 0.88, item 4 0.78, item 5 0.78, item 6 0.66). LMUP scores (median, IQR) were statistically significantly lower in women with ≥3 children (8, 6) compared to women with ≤2 children (11, 2) (p<0.01) and unmarried women who were not in a relationship (7.5, 7) compared to married/ de facto women (11, 2) (p<0.01).

## Discussion

The aim of this study was to evaluate the suitability of the LMUP to determine pregnancy planning in a population of pregnant Australian women. Employing Classical Test Theory our findings show that the tool meets international standards for reliability and validity [41] and confirms this is an appropriate tool measuring pregnancy planning in an Australian antenatal population. Whilst the LMUP is becoming widely used internationally and applied in Australian studies [17, 18], this is the first time the psychometric properties have been evaluated in Australia. With the addition of iodine and vitamin D options in item six, the tool has been adapted and made appropriate for use in the Australian context.

Results of consumer testing through cognitive interviews indicated the tool was understandable and user friendly. These findings, coupled with the lack of missing data indicate the acceptability of the tool. High endorsement (frequent selection of a particular item response option e.g. 90.2% of women reporting they ‘did not use contraception’, Table 1) for the response options of some items was apparent and a limited range of LMUP scores were used. However, it is likely that this has occurred due to the study population including only women attending antenatal care and therefore missing representation of unplanned pregnancies that were terminated who may have selected different response options. This also resulted in negatively skewed data and high planned pregnancy rates in contrast to the original scale development, where the full range of scores were represented. Similar skewed findings were reflected in a recent Dutch antenatal LMUP validation study, with a high percentage of pregnancy planners (84.7% scoring 10–12) [22].

Pregnancy intention is complex and its measurement requires a nuanced and sensitive approach, offered by the LMUP tool [19]. Current international estimates of pregnancy planning measured in population surveys, such as the United States (US) Pregnancy Risk Assessment Monitoring System, are measured simplistically often asking one or two questions. These are sometimes asked up to 5 years after a woman’s last birth, rather than for a current or recent pregnancy, as recommended by the LMUP [28, 42]. This approach includes many limitations including recall bias and potential overestimation of pregnancy intention [28]. A US consensus statement for improving preconception health and care recommend that the LMUP be used as a patient reported outcome [4]. Our successful application of the LMUP both in a clinical maternity setting and online demonstrates how it can be implemented as a simple, quick, reliable tool providing a robust, comprehensive pregnancy intention measure in Australian settings, comparable to international research and outcome measures.

It is important to understand women’s pregnancy planning and intentions, as different approaches to preconception care and engagement are likely needed for women across the spectrum of pregnancy planning [43]. Our study, for example, reflected high rates of pregnancy planning, with 74.4% of women scoring 10–12, classified as planned, 23.7% scoring 4–9 classified as ambivalent and 1.9% were classified as unplanned scoring 0–3, a finding consistent with a prior Australian and international studies [17, 22]. However, despite the large percentage of planned pregnancies overall, item six, which examines the health actions taken in preparation for pregnancy, showed that 28.4% of women took no preconception health actions and 18% only took one preconception health action. This highlights the potential missed opportunity and room for future engagement with women who are planning for pregnancy to improve preconception health by providing them with appropriate support and resources to improve their pregnancy outcomes.

In light of the prevalence of unplanned/ambivalent pregnancies, lack of awareness and poor uptake of preconception health actions, it is critical to consider pregnancy planning to better understand women’s motivations, or lack thereof, for engaging in preconception health and behavior change. Implementation of the LMUP through maternity healthcare (e.g. by midwives or general practitioners) and other healthcare and research settings will provide vital information to raise awareness among policy makers of the breadth and complexity of the issues surrounding pregnancy planning and preconception health in Australia. It will allow researchers and policy makers to further elucidate the characteristics, determinants and social circumstances of women across the continuum of levels of pregnancy planning and engagement. Ongoing application of the LMUP in such a way can provide a population-level indicator for development, evaluation and monitoring of responsive policy, health promotion and primary care interventions to improve pregnancy outcomes. This will inform more sensitive and tailored engagement in improving this area of sexual and reproductive health across the lifespan from universal awareness raising and education to targeted approaches for improved preconception health and pregnancy outcomes.

### Strengths and limitations

This is the first time the LMUP has been adapted and evaluated for use in the Australian context. A strength of this study is the psychometric testing process, including cognitive interviews and a high proportion (42.5%) of women born outside Australia from culturally and linguistically diverse backgrounds. As the current study validated the English version of the Australian LMUP, further studies should also explore whether the LMUP would need to be translated to other language versions. Given that approximately 70% of Australian women birth in public healthcare settings and one-third of Australian women birth in private hospitals, testing the LMUP in both public and private healthcare settings has enabled the study to capture a broad group of Australian women. Whilst the majority of women had post-school education and were from areas of low risk of disadvantage, 30% of women resided in areas at high risk of disadvantage, as defined by the SEIFA criteria [36]. Although the upper age limits for eligible women recruited through public and private settings differ slightly (40 vs 44 years of age respectively), only two women were aged over 40 years. Limitations include testing only in an antenatal population with pregnancies intending to continue to birth; further testing of the LMUP in women with pregnancies which they intend to terminate is warranted.

## Conclusions

The LMUP is a valid and reliable measure of pregnancy planning/intention that can be applied in the Australian population. This study provides evidence of the suitability of the LMUP as a valid and reliable measure of pregnancy planning for Australian pregnant women. Implementation of this tool in maternity care and other healthcare and research settings will provide an accurate population-level estimate of pregnancy intention. Coupled with demographic and contextual information this will reveal the determinants of pregnancy planning/intention to inform improvements in engagement with women to enhance preconception health and reduce adverse birth and longer-term health outcomes. Public health and healthcare professionals such as midwives and general practitioners, researchers and policy makers all play a role in this effort. Importantly, implementation of the LMUP through these settings will enable monitoring and evaluation of the impact of preconception health policies and programs.
